# Dynamic epigenetic changes to *VHL* occur with sunitinib in metastatic clear cell renal cancer

**DOI:** 10.18632/oncotarget.8308

**Published:** 2016-03-23

**Authors:** Grant D. Stewart, Thomas Powles, Christophe Van Neste, Alison Meynert, Fiach O'Mahony, Alexander Laird, Dieter Deforce, Filip Van Nieuwerburgh, Geert Trooskens, Wim Van Criekinge, Tim De Meyer, David J. Harrison

**Affiliations:** ^1^ Edinburgh Urological Cancer Group, Institute of Genetics and Molecular Medicine, University of Edinburgh, Edinburgh, UK; ^2^ Scottish Collaboration On Translational Research into Renal Cell Cancer (SCOTRRCC), Scotland, UK; ^3^ Renal Cancer Unit, The Royal Free Hospital, London, UK; ^4^ Centre for Experimental Cancer Medicine, Bart's Cancer Institute, Queen Mary University of London, London, UK; ^5^ Laboratory of Pharmaceutical Biotechnology, Faculty of Pharmaceutical Sciences, Ghent University, Ghent, Belgium; ^6^ MRC Human Genetics Unit, Institute of Genetics and Molecular Medicine, University of Edinburgh, Edinburgh, UK; ^7^ Biobix: Laboratory of Bioinformatics and Computational Genomics, Department of Mathematical Modeling, Statistics and Bioinformatics, Ghent University, Ghent, Belgium; ^8^ School of Medicine, University of St Andrews, Fife, UK; ^9^ Academic Urology Group, University of Cambridge, Addenbrooke's Hospital, Cambridge Biomedical Campus, Cambridge, UK

**Keywords:** heterogeneity, methylation, mutations, renal cancer, VHL

## Abstract

**Background:**

Genetic intratumoral heterogeneity (ITH) hinders biomarker development in metastatic clear cell renal cancer (mccRCC). Epigenetic relative to genetic ITH or the presence of consistent epigenetic changes following targeted therapy in mccRCC have not been evaluated. The aim of this study was to determine methylome/genetic ITH and to evaluate specific epigenetic and genetic changes associated with sunitinib therapy.

**Patients and methods:**

Multi-region DNA sampling performed on sequential frozen pairs of primary tumor tissue from 14 metastatic ccRCC patients, in the Upfront Sunitinib (SU011248) Therapy Followed by Surgery in Patients with Metastatic Renal Cancer: a Pilot Phase II Study (SuMR; ClinicalTrials.gov identifier: NCT01024205), at presentation (biopsy) and after 3-cycles of 50mg sunitinib (nephrectomy). Untreated biopsy and nephrectomy samples before and after renal artery ligation were controls. Ion Proton sequencing of 48 key ccRCC genes, and MethylCap-seq DNA methylation analysis was performed, data was analysed using the statistical computing environment R.

**Results:**

Unsupervised hierarchical clustering revealed complete methylome clustering of biopsy and three nephrectomy samples for each patient (14/14 patients). For mutational status, untreated biopsy and all treated nephrectomy samples clustered together in 8/13 (61.5%) patients. The only methylation target significantly altered following sunitinib therapy was *VHL* promoter region 7896829 which was hypermethylated with treatment (FDR=0.077, *P*<0.001) and consistent for all patients (pre-treatment 50% patients had VHL mutations, 14% patients VHL hypermethylation). Renal artery ligation did not affect this result. No significant differences in driver or private mutation count was found with sunitinib treatment.

**Conclusions:**

Demonstration of relative methylome homogeneity and consistent *VHL* hypermethylation, after sunitinib, may overcome the hurdle of ITH present at other molecular levels for biomarker research.

## INTRODUCTION

A number of driver genes have been identified in clear cell renal cell cancer (ccRCC) [1–3]; but mutations to the *von Hippel Lindau (VHL)* gene remain the crucial driver mutation in the development of ccRCC [3]. However, mutations of *VHL* and downstream angiogenic genes do not predict response to vascular endothelial growth factor (VEGF) targeted therapy [4]. Indeed none of the established ccRCC driver mutations have been implicated in resistance to targeted therapy. Genetic intratumoral heterogeneity (ITH) in ccRCC is thought to play a role in the evolution of treatment resistance and hinders biomarker development due to inherent variably [3, 5, 6].

*VHL* mutations are identified in 39-85% of sporadic ccRCCs [7–10]; tumors which lack mutations in *VHL* appear to have epigenetic changes or loss of heterozygosity (LOH) at the *VHL* locus [7]. Therefore, inactivation of *VHL* occurs in up to 98% ccRCCs. Recent *in vitro* work by Vanharanta and colleagues has added intriguing functional context to the role of *VHL* in metastatic ccRCC (mccRCC). They identified that activation of metastasis-driving genes downstream of *VHL-HIF*, were enabled by epigenetic events [11]. Therefore dynamic epigenetic changes, particularly to *VHL* and other driver mutations, could contribute to resistance to targeted therapy in mccRCC.

Here we report the effects of sunitinib on mutation and methylation of these driver genes to provide evidence for predictive biomarkers and mechanisms of resistance. For this analysis, sequential ccRCC tumor samples before and after sunitinib therapy, from mccRCC patients within a prospective trial were employed. MethylCap-seq methylation analysis, to detect any highly methylated regions in the genome, with accompanying focused mutation analysis was performed. The study aims were to investigate (i) methylome and genetic ITH; and (ii) consistent epigenetic and genetic changes associated with sunitinib. We hypothesised that: (i) significant methylation changes occur with treatment that may be associated with development of resistance to sunitinib therapy; and (ii) there will be a reduction in private mutations (somatic mutation present in only one of the biopsy or nephrectomy samples for a given patient) following sunitinib therapy due to clonal selection.

## RESULTS

### Patient demographics

DNA from sequential fresh frozen tissue samples was available from 14 patients in the SuMR trial. The characteristics of the patients included in this study are given in Supplementary Table 1 and compared to other patients in the trial from whom sequential tumor DNA was not available.

### Sequencing and methylation summary results

Sequencing of 48 ccRCC genes, related to renal cancer pathogenesis and mechanism of action of agents used in treatment of mccRCC, as listed and detailed in Supplementary Table 2 was performed. The panel was 259.3 Kb in size, contained 1,193 amplicons and gave 98.36% coverage of the submitted genes (Supplementary Table 3 details sequencing summary statistic). The somatic mutations and candidate drivers are listed in Supplementary Table 4 and CNVs relative to normal samples in Supplementary Table 2. In terms of mutations to the commonest ccRCC tumor suppressor genes, baseline mutations (in the untreated samples) were found at the expected frequency, other than for *SETD2* which was higher than expected (expected proportion 11%) [10]: *VHL* mutation in 6 of the 12 patients for whom germline DNA was available (50%; Supplementary Table 5), *PBRM1* in 4 patients (33.3%), *BAP1* in 3 patients (16.7%) and *SETD2* in 6 patients (41.7%). There were no CNVs identified for any of *VHL*, *PBRM1, BAP1* or *SETD2*.

Supplementary Table 6 details the MethylCap-seq data, revealing low (particularly for the biopsies) yet workable coverages for the different samples.

### Hierarchical clustering of methylation and mutational data

**Figure 1:**
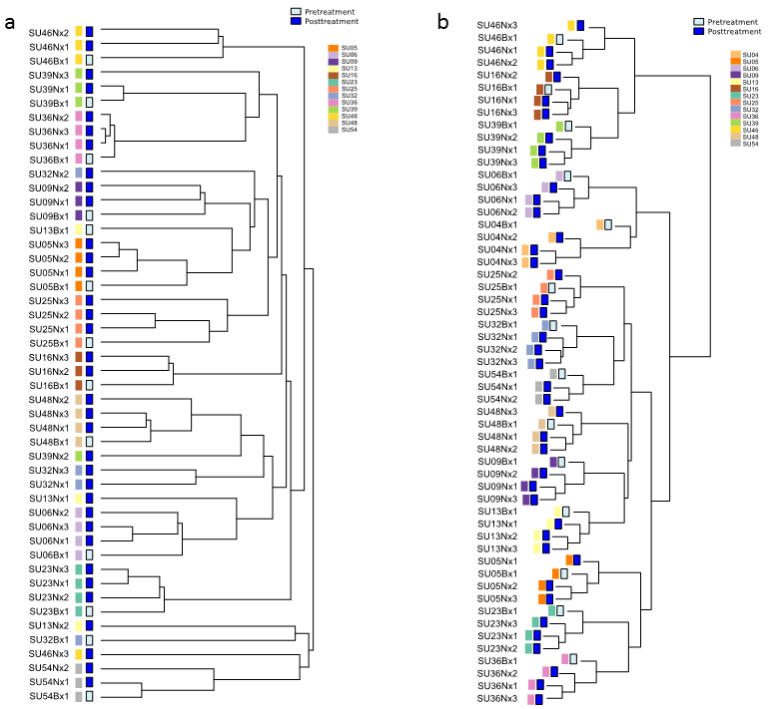
Hierarchical clustering dendrograms of methylation and mutational data **a.** Unsupervised hierarchical clustering of patient sample mutations. 8/13 (61.5%) patient biopsy and nephrectomy samples clustered completely and 4/13 (30.8%) clustered partly together. Supplementary Figure 1 shows the mutational heatmap. **b.** Hierarchical clustering of DNA methylation data. The analysis was performed on 14 matched pairs of untreated (biopsy) and treated (nephrectomy tissue). The 1,000 loci featured by the largest variance (after quantile normalization and log transformation) were used for clustering, employing complete clustering based upon Euclidean distance. For all 14 patients their biopsy and nephrectomy samples were found to cluster. Figure amended from (12) with permission.

Figure 1 shows hierarchical clustering of the 48 key gene mutations (Figure 1a and Supplementary Figure 1) and the 1,000 gene loci featured by the largest methylome variance (Figure 1b), for the biopsy and multiple samples at nephrectomy. As previously shown, the clustering results reveal complete methylome clustering of biopsy and three nephrectomy samples for each individual patient (14/14 patients) [12]. Mutational analysis revealed only 8 of 13 (61.5%) patient's samples clustered. A further 4 of 13 (30.8%) patient samples partly clustered, while there was no clustering in 1 patient sample.

### Methylation differences for targets following sunitinib treatment

**Figure 2:**
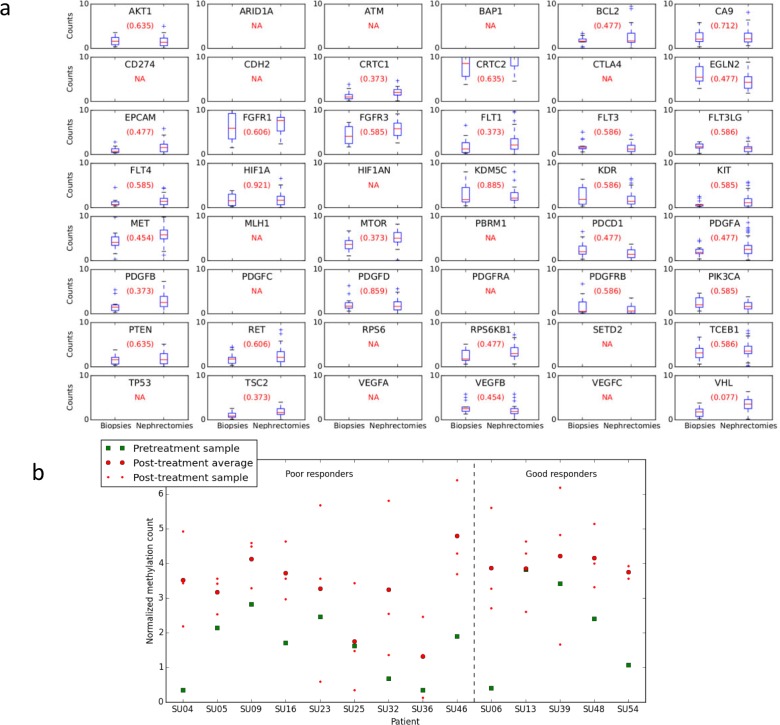
Methylation differences for targets following sunitinib treatment **a.** Comparison of biopsy and nephrectomy for all patients. Target label displayed in each subplot. False discovery rate (FDR) is provided in parenthesis. NA = no methylation core was present, either because the target's regions were filtered due to low average counts, or because no methylation cores were present for the target in the methylome map. If there was more than one region for a certain target, the Figure only shows the most significantly differential region according to *P*-value. *VHL* is the only target that has FDR under the 0.1 significance level (*i.e.* 0.077). The *P*-value is 0.00086 and the logFC -0.8734. The latter implies that the post-treatment samples are more methylated in average than the pre-treatment ones. This is only the case for the methylation core in the *VHL* promoter region 7896829 located from nt 10183068 to nt 10183220 on chromosome 3; other *VHL* regions are not found to be differentially methylated under this significance level. **b.** Per patient methylation of *VHL* at region 7896829. For all samples methylation was greater in the post-treatment nephrectomy samples than the pre-treatment biopsy. Results divided into patients who had a good or poor response to treatment, there was no significant difference in the *VHL* hypermethylation seen in patients with a good *vs* poor response to sunitinib (*P* = 0.896, Student's *t*-test).

We next explored methylation differences for the 48 key target genes following sunitinib treatment (Figure 2a). This supervised approach was preferred to a genome-wide approach that limits the study power, as putatively less informative loci will lead to a substantial increase of the number of hypotheses tested. *VHL* was the only target that has a false discovery rate (FDR) under the 0.1 significance level (FDR = 0.077, *P* < 0.001). The logFC was 0.8734 (Supplementary Table 7) implying that the post-treatment samples were more methylated than the untreated biopsy samples. This was only the case for the methylation core in the *VHL* promoter region 7896829 (located from nt 10183068 to nt 10183220); other *VHL* regions were not significantly differentially methylated.

Taking “Medium to high” and “High to very high” levels of methylation as hypermethylated, 14% of patients had hypermethylation at VHL region 7896829 pre-treatment, and 64% post-treatment (Supplementary Table 5). When assessed on a per-patient basis (Figure 2b), there was variation in baseline *VHL* region 7896829 methylation. The normalized *VHL* methylation level for the biopsy sample had a mean of 1.8 and standard deviation of 1.1. However, for all patients *VHL* methylation at region 7896829 was greater in the post-treatment nephrectomy samples than the pre-treatment biopsy sample. Furthermore, when assessing the response of each patient to sunitinib there was no significant difference in the extent of *VHL* hypermethylation between those patients who had a favourable *vs* poor response to sunitinib (*P* = 0.896, student's *t*-test; Figure 2b).

### Methylation difference for targets following differing sampling conditions

In order to eliminate potential impact of sampling procedure, the *VHL* region was also evaluated in the control hypoxia sample set, taken just prior and following renal artery ligation. There was no effect of sampling identified on *VHL* methylation at location 7896829 (*P* = 0.46, logFC = −0.3151, FDR = 0.76). Furthermore, the negative FC clearly contrasts with the consistently higher methylation degree observed for treated samples, thereby refuting a mere impact of different sample sizes (Supplementary Figure 2). Therefore, it can be concluded that the difference found following sunitinib therapy was not due to the hypoxia effect on the tumor from ligation of the renal artery to fresh sample acquisition. Supplementary Table 8 show for this sample set the methylation differences for all the targets. There is no region that has a significant difference under the FDR < 0.1 significance level.

### Mutation frequency alterations following treatment

**Figure 3:**
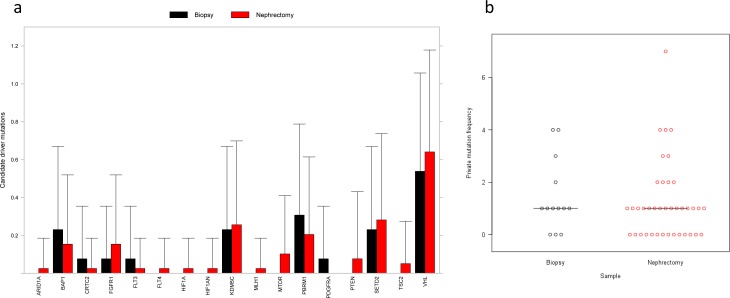
Driver mutation comparison between biopsy and nephrectomy samples **a.** Mean number of SNV/indel candidate driver mutations per gene across all biopsy (15) and nephrectomy (44) samples. Some genes have multiple candidate driver mutations in some samples. Putative passenger somatic mutations are not included. There were no significant differences in mutation count between biopsy and nephrectomy samples (two-sided Wilcoxon rank sum test, *P*≥0.05 for all genes). **b.** Dot plot of private mutation frequency in biopsy and nephrectomy samples. Median value indicated. The number of mutations was greater in the biopsy sample for 7 patients, nephrectomy in 4 samples and equal between biopsy and nephrectomy in 2 samples. There was no significant difference in the number of private mutations in the biopsy samples compared with the median number of private mutations in the nephrectomy samples (*P* = 0.2, unpaired *t*-test).

A comparison of the frequency of candidate driver mutations in the treated and untreated samples showed no significant changes associated with therapy (Figure 3a and Supplementary Table 9). There was no significant change in the number of CNVs found from untreated to treated samples (*P* = 0.57, paired Student *t*-test; Supplementary Table 10), agreeing with the evidence from small mutations. Further analysis showed no significant differences in overall frequency of private mutations (*t*-test, *P* = 0.2) (Figure 3b and Supplementary Table 11) on a per-patient basis, countering the hypothesis that clonal selection will result in a reduction in private mutations.

### Loss of heterozygosity (LOH)

LOH was observed in some samples (supplementary Figure 3, Supplementary Table 12). In these regions, the B-allele frequency (BAF) of germline heterozygous SNPs was clearly shifted away from the expected 0.5, but in no case was it observed to shift entirely to 0 or 1. This could be due to sample purity (normal cells), but given the other observations of ITH, it is more likely to be sub-clonal. In some patient samples LOH was observed in post-treatment samples only, indicating development following therapy (i.e. SU06 chr10), there were no consistent patterns that might account for resistance mechanisms. We observed recurrent LOH of chromosome 3p, consistent with previous reports which showed near universal 3p LOH [3, 5, 13]. Due to the probable sub-clonal nature of the LOH regions detected, we excluded them from further analysis.

### Phylogenetic tree analysis

Phylogenetic trees were generated from the genetic mutation data (CNV, Indel and SNVs) (supplementary Figure 4 and Supplementary Tables 4 and 13). There were truncal mutations in *VHL, PBRM1, BAP1*, *SETD2* in 6/13 tumors. The results showed no consistent inter-patient features for the untreated biopsy samples and a high degree of variability within different tumors supporting the hypothesis of polyclonal evolution in mRCC and little effect of treatment on this process.

## DISCUSSION

In this study, using a rare sample set of matched fresh frozen pre- and post-sunitinib treated ccRCC samples, dynamic epigenetic changes to *VHL* were identified. There was also complete epigenetic clustering of all samples taken from the same patients, suggesting relative homogeneity of the methylome. Together these findings suggest that consistent methylation alterations may be an attractive field for biomarker research.

In order to evaluate the potential of DNA methylation as a more stable readout in the process of response and acquisition of resistance to targeted therapy, the change in methylation status of the 48 ccRCC related genes were evaluated. A genome-wide approach could have been performed but limits the power, as putatively less informative loci will lead to a substantial increase of the number of hypotheses tested. Interestingly, *VHL* methylation status was the sole gene, of the 48 evaluated, which was significantly altered following sunitinib treatment. *VHL* hypermethylation was consistent when considering all biopsy *vs* all nephrectomy samples and also on a per-patient basis. The finding of significant changes to *VHL*, which is intrinsic to ccRCC, underlines the potential of this platform for future research.

It has been established that the procurement conditions for fresh tumor tissue can greatly affect gene and protein expression [14]. Therefore we collected two paired control samples (biopsy and nephrectomy) in which no sunitinib was given to ensure these findings were not purely representative of the subtle differences in sample collection. No significant changes to *VHL* methylation were seen in these 2 pairs implicating sunitinib. A lack of power is an unlikely alternative explanation, as the non-significant effect was in a different direction to that consistently associated with sunitinib treatment.

It is likely that the change to *VHL* methylation is due to sunitinib rather than progression of the disease; agreeing with previous work refuting a link between *VHL* inactivation and aggressive ccRCC [8]. Analysis of biopsy tissue prior to sunitinib showed a high degree of *VHL* methylation variability. A relatively short period of treatment resulted in an increase in methylation in all samples, implicating a treatment effect. This finding is supported by preclinical and clinical data also demonstrating hypermethylation and downregulation of key tumor suppressor genes (*PTEN*) in leukaemia and GISTs [15–17]. Vanharanta *et al* analyzed metastatic subpopulations of *VHL*-deficient ccRCC cells and demonstrated that during ccRCC progression there were epigenetic alterations in the *VHL-HIF* pathway, these alterations were associated with metastasis and a poor prognosis [11]. In congruity with the work of Vanharanta, the data presented in this manuscript, show consistent *VHL* hypermethylation (above any baseline *VHL* methylation levels) following a 18-week period of treatment with sunitinib. This consistent molecular change allows further hypotheses to be developed with regard to predictive ability of *VHL* methylation and also the role of *VHL* methylation in the development of acquired resistance to sunitinib.

Genetic mutational analysis showed clustering of pre-treatment biopsy and all post-treatment nephrectomy samples only occurred in 61.5% patients. Additionally, the phylogenetic trees illustrate relative homogeneity of mutation status between biopsy and nephrectomy samples, but variability between samples from the same patient. Furthermore, LOH analysis showed the presence of LOH across the sample set, but at a subclonal level only and without consistency between untreated and treated samples. As such, there was genetic ITH when considering the somatic mutations (SNPs, indels, and CNVs) found in 48 key ccRCC genes in the biopsy and multi-region sampling of the post-treatment nephrectomy sample. Assessment of driver mutations alone, revealed no significant differences in mutation counts between biopsy and nephrectomy samples. These data confirm genetic ITH and a failure to identify consistent genetic biomarkers in mccRCC, as we and others have demonstrated previously [3, 6]. It was hypothesized that there would be a reduction in private mutations following sunitinib therapy, due to a process of clonal selection. This hypothesis was not proven, as private mutation status was comparable before and after sunitinib treatment. As such, the 18-week sunitinib treatment period does not have an obvious effect on private mutation frequency and has little effect on the baseline level of genetic ITH. It may be that genetic clonal evolution with sunitinib takes longer to develop and the tissue collection at 18 weeks was premature.

This study has a number of important strengths, including: access to a very rare sample set of matched pre- and post-sunitinib treatment ccRCC samples (meaning validation was not possible), control samples to ensure renal artery ligation was not responsible for the methylation results identified, and the high throughput analyses employed. However, we are also aware of the study shortcomings: the specific timing of sunitinib treatment, limitations of a single biopsy *versus* the multiregion sampling of the nephrectomy specimen, and targeted sequencing of only 48 genes preventing direct comparisons of mutational and methylation clustering. The significant result for *VHL* illustrates that the limited MethylCap-seq coverages were still workable. The collection of sequential tumor tissue for epigenetic analysis has proven challenging in RCC. This is reflected by the lack of literature in this setting or on methylation as a potential biomarker in mccRCC. Our work aimed to explore if the dynamic epigenetic changes with sunitinib were as complex and variable as those seen with DNA, and was successful in that respect. It was not powered to define predictive biomarkers, which would require hundreds of patients each with potentially multi-region tumor sampling. A crude comparison of responders and non-responded showed no difference in *VHL* methylation levels following therapy. Whether these findings would apply at different time points or at progression should be the focus of future work.

There is evidence from TGCA data to show biological relevance to the *VHL* methylation demonstrated in this study. There is no TCGA data (based on Infinium HumanMethylation microarrays) on ccRCC for the region picked up by our analysis, (www.mexpress.be [VHL gene symbol, KIRC dataset]). However, when looking at other tumors (profiled using the more recent HM450 version of the array, e.g. in colon adenocarcinoma dataset, COAD, HM450 data for a subset of the tumors), 3 probes targeting the *VHL* region of described in our work (i.e. cg10352003, cg06034437 and cg01998262), exhibit clear methylation. Moreover, for the middle probe there is proof of a significant (negative) association between methylation and expression (COAD dataset); furthermore, this association is the strongest of all VHL probes. Finally, for cg10352003, differential methylation was already demonstrated in a prostate cancer context [18]. The data presented in the current study showed significant consistently increases in *VHL* methylation in all 13 paired samples after a short period of sunitinib. In view of the mode of action of sunitinib and the pathogenesis of ccRCC this finding is likely to have mechanistic importance in our understanding of the effect of sunitinib at a molecular level. These findings occurred in responders and non-responders alike, suggesting a more global effect on the tumor rather than a predictive type biomarker. One might expect that those patients with functional *VHL* may develop hypermethylation as a mechanism of resistance to sunitinib. However, *VHL* hypermethylation with sunitinib occurred in patients with wild-type and those with mutant *VHL*; thus, one can speculate that *VHL* hypermethylation is a mechanism of global resistance rather than a unique phenomenon associated with rare cases where *VHL* is not altered. However larger studies with multiple biopsies at more time points would be required to define predictive biomarkers. It may be that biopsies taken at progression, as well as after an initial period of therapy, gives better insight into the biology of treatment failure.

Our findings provide insight into the dynamic and consistent epigenetic changes occurring with sunitinib. The hurdles that genetic, transcriptomic and proteomic heterogeneity provide to biomarker exploration in ccRCC may be overcome by exploiting the stability of the methylome.

## MATERIALS AND METHODS

### Patients and samples

Primary tumor tissue (the “sunitinib set”) was collected and snap frozen from mccRCC patients treated with three cycles of pre-surgical sunitinib (Sutent™, Pfizer, Sandwich, UK), for 18 weeks, as a translational endpoint of a previously reported prospective clinical trial (SuMR; NCT01024205) [19]. The clinical trial and subsequent genetic analysis has appropriate ethical approval (REC: 07/Q0603/58). Patient matched tissue was taken at two time points from the primary renal tumor (at baseline and after 18 weeks of therapy), the latter at the time of cytoreductive nephrectomy. As described previously, to address ITH, multiple spatially separate tumor samples (*n* = 3) were taken at the 2^nd^ time point approximately 30 minutes following ligation of the renal artery [6]. Thus, for each patient there were intended to be four cancer samples. Normal renal tissue was also sampled to provide normal genomic DNA.

To evaluate the effect of tumor hypoxia on molecular changes, an additional patient matched sample set (the “hypoxia set”) was obtained from two patients. These samples were obtained as part of the Scottish Collaboration On Translational Research into Renal Cell Cancer (SCOTRRCC) study (East of Scotland Research Ethics Service REC 1: 10/S1402/33). These patients, who were undergoing open cytoreductive nephrectomy for mccRCC, had fresh primary ccRCC tumour biopsies taken prior to ligation of the renal artery and then further matched fresh frozen tumor samples harvested following ligation and division of the renal artery and removal of the kidney. This hypoxic set acted as a control for the sampling methodology.

### DNA extraction

Frozen section histology was performed to ensure that the tissue used for DNA extraction contained viable ccRCC tissue. Genomic DNA extraction was undertaken using the Qiagen DNeasy Blood and Tissue (Qiagen, Manchester, UK) kit as per the manufacturer's instructions.

### Sequencing

A custom Ampliseq panel (Thermo Fisher Scientific, Paisley, UK) was designed to assess 48 key ccRCC genes in 63 samples across 13 patients for which DNA samples remained following the methylome analysis. Eleven patients had all five associated samples (normal, biopsy (Bx), and three post-treatment nephrectomy (Nx)), one patient (SU16) had all but the normal sample, and one patient (SU54) had only two nephrectomy samples. Multiplex PCRs were performed according to the manufacturer's instructions across two primer pools. The samples were sequenced on an Ion Proton sequencer to a mean on-target depth of 4000X with 97-98% of bases over 15X. Samples were aligned to the hg19 human genome reference assembly and variants identified with TorrentSuite 4.2 (Thermo Fisher Scientific). Sequencing data is available *via* NCBI SRA (Accession number SRP056914).

### Variant filtering and annotation

Variants were filtered to exclude those with genotype quality < 60 or variant allele frequency < 10% (summed over all alternate alleles), or located within 1bp of a homopolymer run of at least 4bp. The remaining variants were grouped by patient and classified by the degree of sharing of genotypes (Supplementary Table 13). Variants with non-reference genotypes occurring only in the tumour samples for a given patient, and not occurring in any normal samples across the patient set were considered tumour-specific somatic mutations. Ensembl Variant Effect Predictor [20] was used to annotate the somatic mutations. Frameshift, in-frame deletion, missense, nonsense (stop gained), splice acceptor, and splice donor mutations were considered candidate driver mutations. Missense mutations predicted to be at least possibly or probably damaging by at least one of SIFT [21] or Polyphen 2 [22] were also considered candidate driver mutations.

### Copy number variation

EXCAVATOR 2.2 [23] was modified to handle targeted sequencing input and used to infer copy number variants by comparing normalized target coverage between the tumour (biopsy and nephrectomy) and normal samples. CNVs were identified for the 12 patients with a normal sample (excluding SU16) (Supplementary Table 13).

### Loss of heterozygosity

Bi-allelic SNPs that were clearly heterozygous (B allele frequency (BAF) between 0.4 and 0.6 inclusive) in the normal sample for a patient were selected (excluding SU16). Reads supporting each allele were counted directly from the BAM files for all samples for the patient. BAFs for the tumour samples were calculated and plotted in R, and ExomeCNV [24] was used to identify regions of LOH (Supplementary Table 12).

### Clustering and phylogenetic trees

Samples were clustered according to their somatic mutations (SNPs, indels, and CNVs) using the R packages ape 3.1-1 and igraph 0.7.1 [25, 26]. For each individual patient, we calculated a Manhattan distance matrix and generated a phylogenetic tree using the bionj algorithm implemented in the R package ape [25, 27]. Branches were labelled with candidate driver mutations (SNPs, indels, and CNVs) that were consistent with the tree.

### DNA methylation analysis

DNA methylation analysis (MethylCap-seq) was performed as outlined previously [28], except that solely the MethylCap kit (Diagenode, Liège, Belgium) was used for capturing methylated fragments from 500ng starting material and that massively parallel sequencing of these fragments was subsequently performed on the Illumina HiSeq 2000 (Illumina, San Diego, CA, USA). Methylation data is available *via* GEO (Accession number GSE67700).

### Methylation data processing and analysis

Raw data files were mapped with BOWTIE on human reference genome Hg19/GRCh37, and summarized using an in-house developed Map of the Human Methylome (http://www.biobix.be/map-of-the-human-methylome/mhm-version-2/) consisting of a putatively genome-wide overview of potentially methylated loci (“methylation cores”). Further data analysis was performed with Python 3.4.1 and R 3.0.1. The Bioconductor LIMMA package (3.16) was used to identify regions featured by differential methylation (applying quantile normalization). LIMMA was originally a library for microarray analysis, but the voom function that was used also allows for sequencing count data analysis, and is particularly suitable in case of library size differences [29].

For both the main and the hypoxia dataset, only methylation cores that referred to annotated promoter regions (including exon1) and had at least an average coverage of one mapped fragment per core were used for analysis with voom and LIMMA. Low coverage loci are featured by insufficient power to be detected as differentially methylated and were removed from the final dataset to avoid inflation of the number of hypotheses tested. The final fit to determine differential methylation was obtained with the LIMMA functions lmFit and eBayes. Methylation cores corresponding to the target genes were subsequently selected for *P*-value and FDR estimation (Benjamini-Hochberg), an FDR significance threshold of 10% was selected.

For the 48 key targets, a per-patient analysis was also performed. First, the quantile segments of all the normalized counts were determined for the region, in order to have four categories of counts: ‘No to low methylation’, ‘Low to medium’, ‘Medium to high’, and, ‘High to very high’. Then, for each patient, the pre- and post-treatment condition was determined based on their average methylation count. ‘Medium to high’ and ‘High to very high’ were both taken as hypermethylated state.

The methylation data was also used to for an unsupervised hierarchical clustering analysis of the samples. The 1,000 loci featured by the largest variance (after quantile normalization and log transformation) were used for clustering, employing complete clustering based upon Euclidean distance.

### Statistical analysis

Unpaired student *T*-test and Chi-squared test were used to compare continuous and categorical data respectively.

## SUPPLEMENTARY MATERIALS FIGURES AND TABLES




