# Supplementary material for: Dynamic epigenetic changes to VHL occur with sunitinib in metastatic clear cell renal cancer
Source: Oncotarget. 2016 Mar 23;7(18):25241–50. doi: 10.18632/oncotarget.8308 (PMC5041900; doi:10.18632/oncotarget.8308)
Supplement: Supplementary file 1 [file oncotarget-07-25241-s001.pdf]

Dynamic epigenetic changes to *VHL* occur with sunitinib in metastatic clear cell renal cancer

Supplementary Material

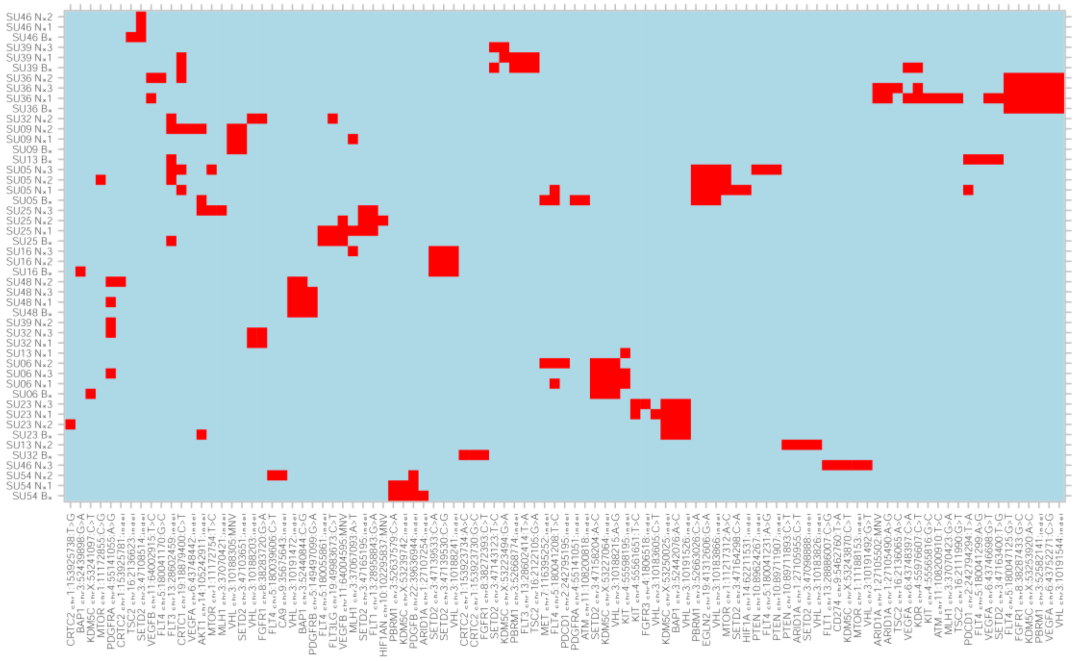

Supplementary Figure 1. Unsupervised clustering of patient DNA samples by somatic mutation status. Gene name for all somatic mutations and ref/alt alleles for SNV are given. The name of the variant type of MNVs/indels are specified. Mutational analysis revealed 8 of 13 (61.5%) patients samples clustered. A further 4 of 13 (30.8%) patient samples partly clustered, while there was no clustering in 1 patient sample.

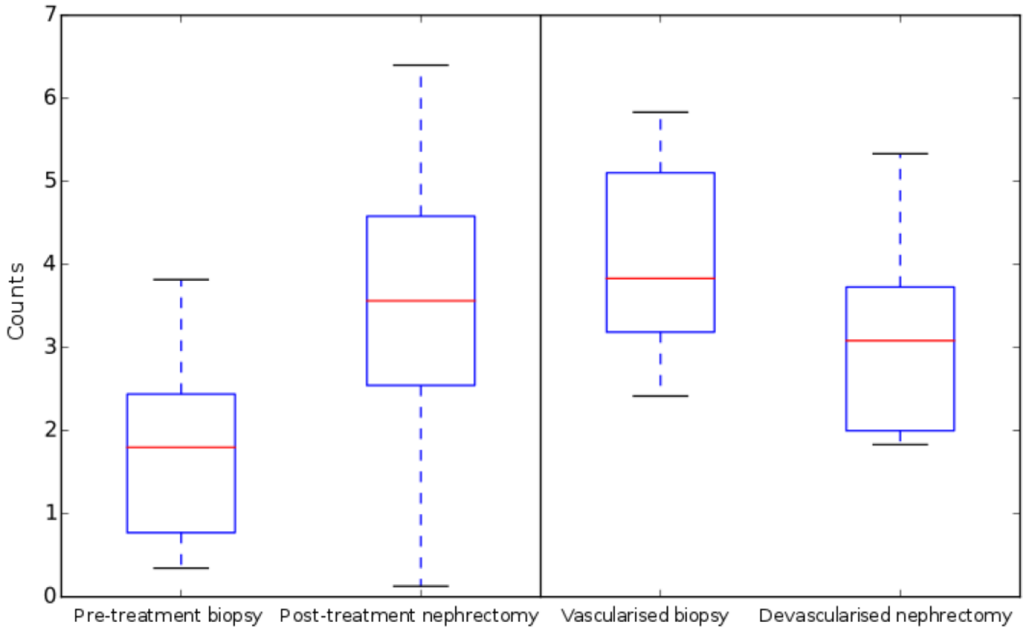

Supplementary Figure 2. Box plots of normalized counts for *VHL* region 7896829 in sunitinib and hypoxia sample sets. Methylation of *VHL* region 7896829 was significantly increased in the post-sunitinib treated nephrectomy samples (*P*-value<0.001, logFC=0.8734; FDR=0.077,). Whereas, there was no significant change in methylation in *VHL* region 7896829 (*P*-value=0.46, logFC=-0.3151, FDR=0.76) following clamping of the renal artery (devascularised nephrectomy sample). Indicating no effect of sampling on methylation of *VHL* region 789629.

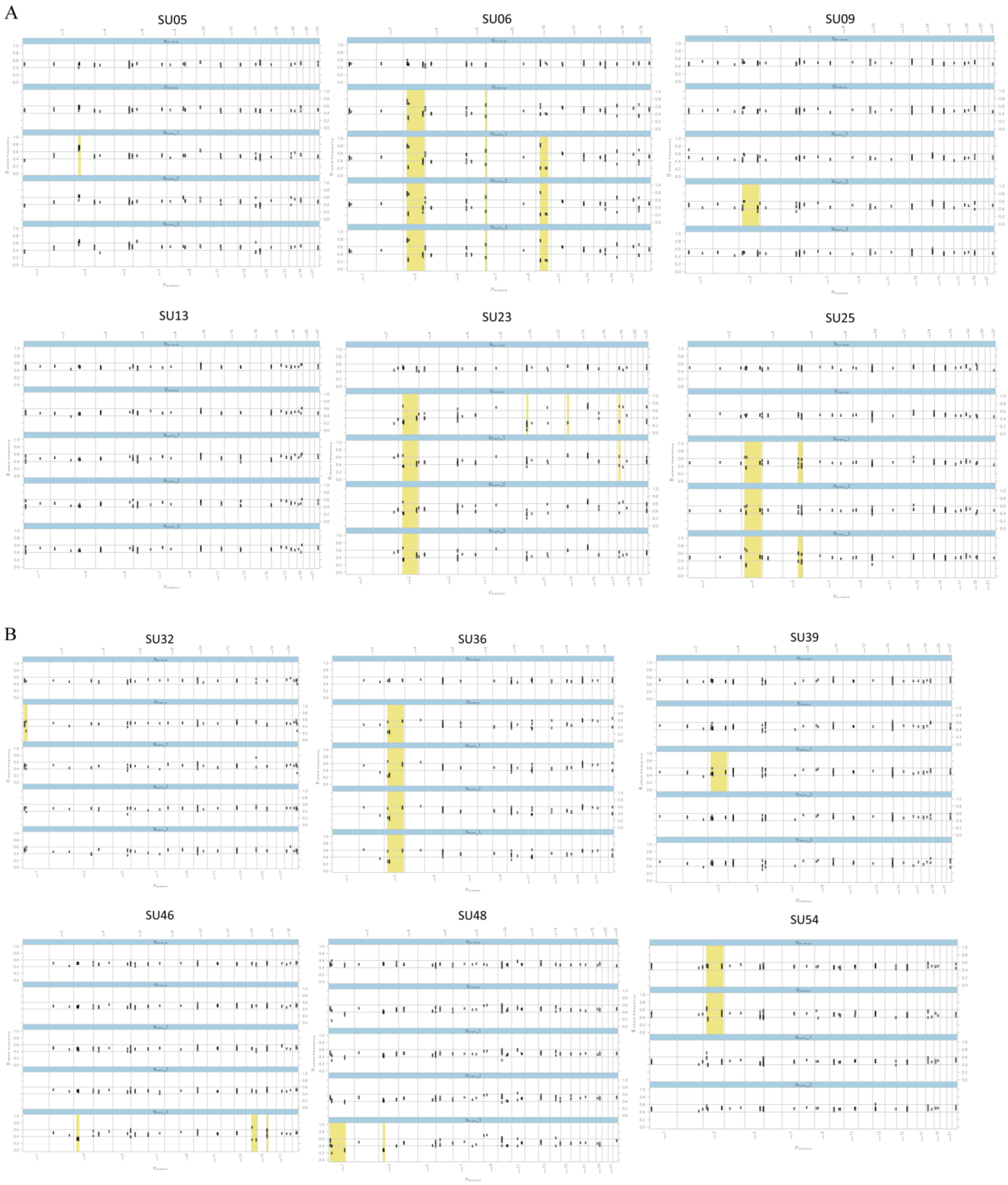

Supplementary figure 3. Loss of heterozygosity plots. (a) Patients SU05-SU25, (b) patients SU32-54. LOH occurring in all cells in a sample would result in germline heterozygous sites in the region covered by entirely reference or entirely alternate reads, which is evidenced by a B allele frequency (BAF: # alternate allele reads/total

reads) of 0 or 1. An incomplete shift of the BAF away from 0.5 towards 0 or 1 indicates that not all cells in the sample have lost one copy of the locus (highlighted regions). This is no plot for sample SU16 as there was no normal sample from which to derive LOH.

A

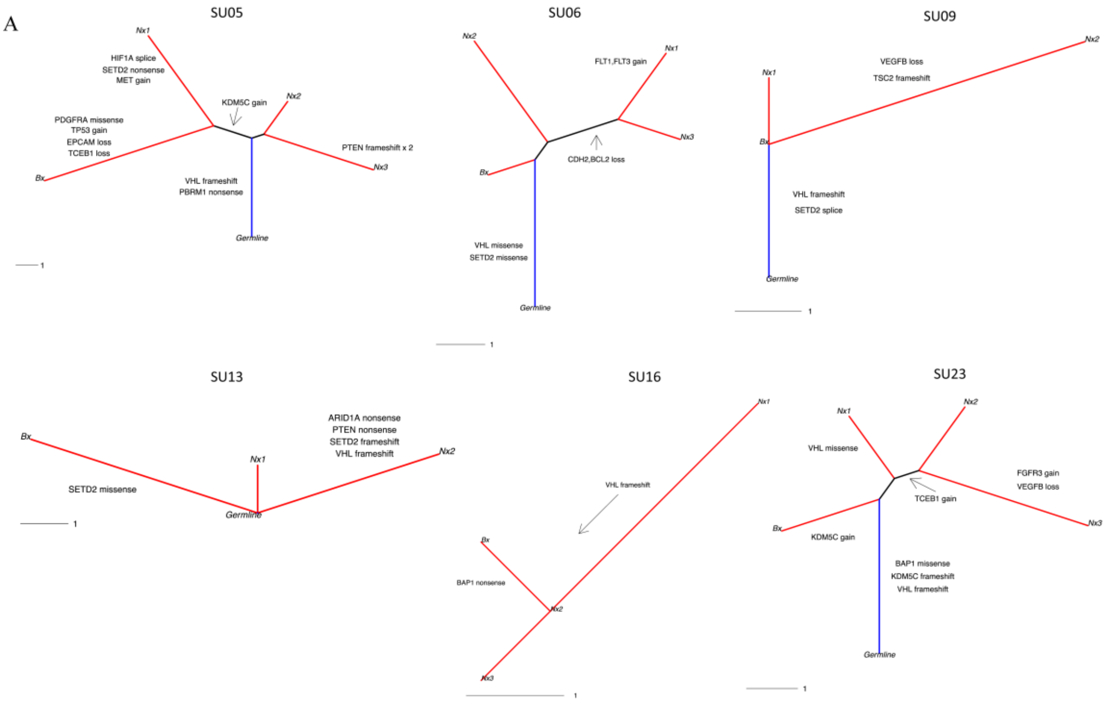

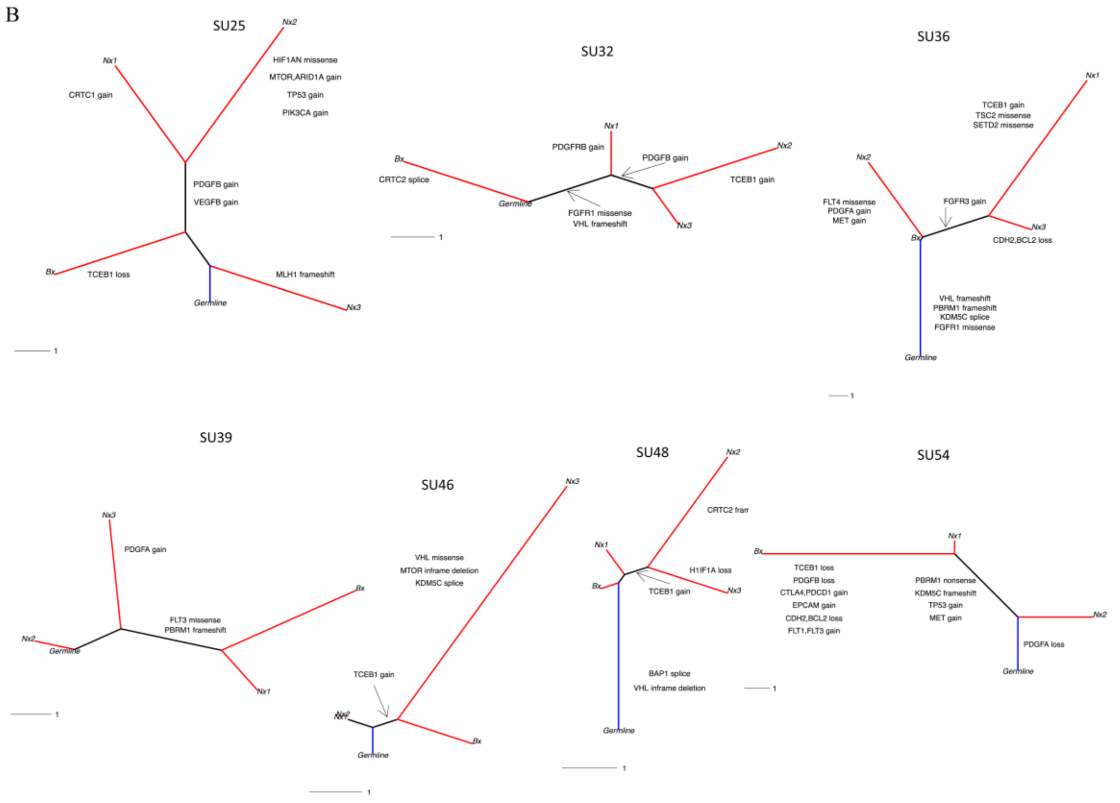

Supplementary figure 4. Phylogenetic trees based on SNPs, indels and CNVs created using neighbour joining. (a) Patients SU05-SU23, (b) patients SU25-54. The branch lengths include passenger mutations as well as drivers. Branch and trunk length are proportional to the number of mutations acquired on the corresponding branch or trunk (blue line for germline trunk edges, black for internal edges, and red for private mutations). Driver mutations were acquired by the indicated genes in the branches the arrows indicate. For branch with no labels, there were only passenger mutations defining that branch. Trees are rooted at the germline DNA sequence, determined by sequencing of DNA from normal renal tissue. These trees demonstrate that despite relative homogeneity of mutation status between biopsy and nephrectomy samples there is variability between samples from the same patient. The scale bar shows the branch length corresponding to 1 mutation.

Supplementary table 1. Patient characteristics of those patients from whom tumor DNA was available for analysis from sequential tissue. A comparison with the entire SuMR trial cohort is provided.

	**Sequential frozen tissue cohort**	**Cohort enrolled into clinical trial**	***P*-value**
Number of patients	14	43	N/A
Age, median (range)	67 (52-78)	61 (38-78)	0.12
Male gender (%)	11 (78.6)	34 (79.1)	0.97
Baseline VHL mutation (%)	6/12 (50)[+]	NA	N/A
Baseline VHL hypermethylation status (%)	2/14 (14)	NA	N/A
MSKCC prognostic risk [13] (%)			0.22
Intermediate	11 (78.6)	26 (60.4)	
Poor	3 (21.4)	17 (39.5)	
Metastatic sites (%)			0.94
1-2	10 (71.4)	31 (72.1)	
3+	4 (28.6)	13 (30.2)	
Clear cell tumour grade, identified at nephrectomy (%)*			0.76
1-2	7 (50)	14/31 (45.2)	
3-4	7 (50)	17/31 (54.8)	
Median PFS, months (95% CI)	17.5 (7-21)	6.8 (4.9-19)	0.03

N/A-not applicable; MSKCC-Memorial Sloan Kettering Cancer Center; PFS-progression free survival; NA-not available; CI-confidence interval; *only 31 of the

43 patients in the SuMR study had a cytoreductive nephrectomy; [+]denominator of 12 patients as there was not adequate DNA from SU04 for mutation analysis following methylation studies and SU16 did not have a germline specimen with which to compare

Supplementary table 2. 48 key ccRCC genes analysed in this study. A full coordinate list in hg19/GRCh37 with Ensembl gene ids is provided. Genes were decided upon due to their role in: ccRCC pathogenesis; tyrosine kinase inhibitor (TKI) or other ccRCC treatment activity (mTOR inhibitor or T-cell checkpoint inhibitors); ccRCC driver mutations [14–16]; VHL downstream targets; or unpublished in house experimental candidates.

Gene	Rationale for study	Ensembl ID	Chromosome	Start	End	Strand
MTOR	Driver	ENSG00000198793	1	11166592	11322564	-1
ARID1A	Driver	ENSG00000117713	1	27022524	27108595	1
CRTC2	mTOR inhibitor target	ENSG00000160741	1	153920145	153931101	-1
EPCAM	Unpublished work	ENSG00000119888	2	47572297	47614740	1
CTLA4	Immune pathways	ENSG00000163599	2	204732509	204738683	1
PDCD1	Immune pathways	ENSG00000188389	2	242792033	242801060	-1
VHL	Driver	ENSG00000134086	3	10182692	10193904	1
MLH1	Unpublished work	ENSG00000076242	3	37034823	37107380	1
SETD2	Driver	ENSG00000181555	3	47057919	47205457	-1
BAP1	Driver	ENSG00000163930	3	52435029	52444366	-1
PBRM1	Driver	ENSG00000163939	3	52579368	52719933	-1
PIK3CA	Driver	ENSG00000121879	3	178865902	178957881	1
FGFR3	TKI	ENSG00000068078	4	1795034	1810599	1
PDGFRA	TKI	ENSG00000134853	4	55095264	55164414	1
KIT	TKI	ENSG00000157404	4	55524085	55606881	1
KDR	TKI	ENSG00000128052	4	55944644	55991756	-1
PDGFC	TKI	ENSG00000145431	4	157681606	157892546	-1
VEGFC	TKI	ENSG00000150630	4	177604689	177713881	-1
PDGFRB	TKI	ENSG00000113721	5	149493400	149535435	-1
FLT4	TKI	ENSG00000037280	5	180028506	180076624	-1
VEGFA	TKI	ENSG00000112715	6	43737921	43754224	1
PDGFA	TKI	ENSG00000197461	7	536895	559933	-1
MET	MET inhibitor	ENSG00000105976	7	116312444	116438440	1
FGFR1	TKI	ENSG00000077782	8	38268656	38326352	-1
TCEB1	Driver	ENSG00000154582	8	74851404	74884522	-1
CD274	Immune pathways	ENSG00000120217	9	5450503	5470566	1
RPS6	mTOR inhibitor	ENSG00000137154	9	19375713	19380252	-1
CA9	VHL downstream	ENSG00000107159	9	35673853	35681156	1
RET	TKI	ENSG00000165731	10	43572475	43625799	1
PTEN	mTOR inhibitor	ENSG00000171862	10	89622870	89731687	1
HIF1AN	VHL downstream	ENSG00000166135	10	102288829	102319755	1
VEGFB	TKI	ENSG00000173511	11	64002010	64006259	1
PDGFD	TKI	ENSG00000170962	11	103777914	104035107	-1
ATM	Driver	ENSG00000149311	11	108093211	108239829	1
FLT3	TKI	ENSG00000122025	13	28577411	28674729	-1
FLT1	TKI	ENSG00000102755	13	28874489	29069265	-1
HIF1A	VHL downstream	ENSG00000100644	14	62162231	62214976	1
AKT	mTOR inhibitor	ENSG00000142208	14	105235686	105262088	-1
TSC2	mTOR inhibitor	ENSG00000103197	16	2097466	2138716	1
TP53	Driver	ENSG00000141510	17	7565097	7590856	-1

RPS6KB1	mTOR inhibitor	ENSG00000108443	17	57970447	58027925	1
CDH2	Unpublished work	ENSG00000170558	18	25530930	25757410	-1
BCL2	Unpublished work	ENSG00000171791	18	60790579	60987361	-1
CRTC1	mTOR inhibitor	ENSG00000105662	19	18794487	18893004	1
EGLN2	VHL downstream	ENSG00000269858	19	41304901	41314338	1
FLT3LG	Immune pathways	ENSG00000090554	19	49977464	49989488	1
PDGFB	TKI	ENSG00000100311	22	39619364	39640756	-1
KDM5C	Driver	ENSG00000126012	X	53220503	53254604	-1

Supplementary table 3. Summary statistics from Ion Proton sequencing. Total target length 259.3 Kb. Uniformity is defined as the percentage of bases in all targeted regions covered by at least 0.2x the average base coverage depth. Variants include SNVs and indels.

Patient	Sample	Mapped Reads	On Target	Mean Depth	Uniformity	Variants
SU05	Normal	6,260,610	92.78%	4,193	91.88%	186
SU05	Bx	4,584,787	94.10%	2,882	86.12%	204
SU05	Nx1	7,320,935	92.97%	4,402	86.47%	213
SU05	Nx2	9,216,597	90.03%	5,623	88.95%	211
SU05	Nx3	12,782,714	92.33%	8,120	90.18%	213
SU06	Normal	3,565,245	96.21%	2,530	91.70%	177
SU06	Bx	5,919,756	97.05%	4,162	91.57%	180
SU06	Nx1	5,906,200	95.06%	4,025	91.69%	184
SU06	Nx2	6,290,951	96.34%	4,062	84.79%	215
SU06	Nx3	5,676,828	96.73%	3,985	90.40%	182
SU09	Normal	4,755,746	97.07%	3,363	90.97%	172
SU09	Bx	5,602,633	96.43%	3,907	91.01%	177
SU09	Nx1	4,552,874	96.30%	3,193	91.85%	182
SU09	Nx2	9,099,484	96.72%	5,972	88.89%	202
SU09	Nx3	6,923,975	96.99%	4,890	90.90%	172
SU13	Normal	4,517,736	96.81%	3,192	91.29%	171
SU13	Bx	7,371,058	94.53%	4,627	85.86%	227
SU13	Nx1	4,638,491	91.93%	3,057	91.35%	174
SU13	Nx2	6,585,640	96.38%	4,588	90.52%	177

SU13	Nx3	4,612,575	95.23%	3,196	91.79%	175
SU16	Bx	5,432,470	97.22%	3,798	91.37%	200
SU16	Nx1	4,628,700	95.28%	3,262	89.68%	192
SU16	Nx2	4,527,569	96.34%	3,151	91.11%	192
SU16	Nx3	5,749,936	97.32%	4,047	91.48%	189
SU23	Normal	5,470,650	96.67%	3,880	87.92%	180
SU23	Bx	3,752,510	96.25%	2,610	88.91%	173
SU23	Nx1	8,153,665	97.48%	5,812	89.38%	181
SU23	Nx2	3,454,766	95.09%	2,408	86.21%	169
SU23	Nx3	3,876,524	97.19%	2,749	91.38%	179
SU25	Normal	5,583,548	94.18%	3,455	86.52%	246
SU25	Bx	6,429,958	97.44%	4,579	90.46%	214
SU25	Nx1	5,326,845	97.09%	3,777	89.37%	211
SU25	Nx2	4,254,782	96.98%	2,987	88.98%	204
SU25	Nx3	6,076,824	93.23%	3,703	86.37%	244
SU32	Normal	4,786,538	92.19%	2,886	86.55%	208
SU32	Bx	4,700,290	93.04%	2,768	83.99%	199
SU32	Nx1	9,789,195	96.36%	6,423	87.64%	204
SU32	Nx2	5,170,852	96.99%	3,614	90.45%	185
SU32	Nx3	3,627,027	93.63%	2,216	84.83%	188
SU36	Normal	7,596,453	95.39%	5,061	85.23%	192
SU36	Bx	4,612,502	96.04%	3,021	85.90%	189
SU36	Nx1	3,340,867	94.82%	1,971	82.12%	258
SU36	Nx2	5,584,816	95.40%	3,463	85.05%	217

SU36	Nx3	2,638,106	94.27%	1,583	84.05%	227
SU39	Normal	5,306,254	95.96%	3,726	89.81%	189
SU39	Bx	5,418,980	94.39%	3,310	85.39%	245
SU39	Nx1	7,235,469	96.95%	5,082	88.72%	197
SU39	Nx2	5,053,700	96.71%	3,546	90.05%	192
SU39	Nx3	3,232,085	89.79%	1,861	84.00%	234
SU46	Normal	7,835,431	97.05%	5,588	90.94%	200
SU46	Bx	7,753,905	76.82%	4,244	90.52%	193
SU46	Nx1	5,301,729	83.44%	3,188	91.00%	194
SU46	Nx2	5,444,644	81.32%	3,170	90.18%	188
SU46	Nx3	7,445,806	90.86%	4,855	89.47%	200
SU48	Normal	8,904,430	96.52%	6,328	89.57%	181
SU48	Bx	7,224,100	90.19%	4,753	90.16%	181
SU48	Nx1	7,739,889	97.45%	5,492	89.24%	186
SU48	Nx2	5,932,421	96.87%	4,187	88.98%	176
SU48	Nx3	7,114,336	84.55%	4,363	90.83%	179
SU54	Normal	7,787,869	96.44%	4,683	75.25%	296
SU54	Bx	4,640,094	92.65%	3,077	86.87%	214
SU54	Nx1	5,693,458	96.83%	3,923	88.55%	210
SU54	Nx2	7,762,492	95.68%	5,415	90.91%	211

Supplementary Table 4. Excel file detailing the somatic mutations identified on a per-patient basis, candidate driver mutations are indicated.

Supplementary table 5. *VHL* mutational and hypermethylation status at biopsy and post-sunitinib nephrectomy. *VHL* hypermethylation status was taken as those patient samples with "medium to high" and "high to very high" levels of methylation at *VHL* region 7896829. For *VHL* mutational status the denominator was 12 patients as there was not adequate DNA from SU04 for mutation analysis following methylation studies and SU16 did not have a germline specimen with which to compare. Damaging mutations to *VHL* were included (frameshifts; in-frame insertions and deletions; nonsense mutations and missense mutations predicted to be damaging).

	***VHL* mutation**		***VHL* hypermethylation**	
**Patient**	**Biopsy**	**Nephrectomy**	**Biopsy**	**Nephrectomy**
SU04	N/A	N/A	N	Y
SU05	Y	Y	N	N
SU06	Y	Y	N	Y
SU09	Y	Y	N	Y
SU13	N	Y	Y	Y
SU16	N/A	N/A	N	Y
SU23	Y	Y	N	N
SU25	N	N	N	N
SU32	N	Y	N	N
SU36	Y	Y	N	N
SU39	N	N	Y	Y
SU46	N	Y	N	Y
SU48	Y	Y	N	Y
SU54	N	N	N	Y
**Total**	6/12 (50%)	9/12 (75%)	2/14 (14%)	9/14 (64%)

Supplementary table 6. MethylCap-seq study characteristics. Columns represent coverage (quantity of sequenced paired-end fragments), amount and fraction of mapped fragments, and final library sizes of data used for statistical analysis.

**SuMR samples**				
**Sample name**	**Coverage**	**Mapped reads**	**Mapping percentage (%)**	**Library size**
SU04 Bx	9.54E+06	4448158	46.61	2091534
SU04 Nx1	1.91E+07	10382230	54.48	5898729
SU04 Nx2	1.26E+07	6878511	54.51	3820017
SU04 Nx3	1.04E+07	6107566	58.64	2774339
SU05 Bx	7.27E+06	3352838	46.14	1612202
SU05 Nx1	9.87E+06	6035692	61.14	2679421
SU05 Nx2	1.51E+07	9023031	59.62	4067148
SU05 Nx3	1.52E+07	8673515	56.89	3954518
SU06 Bx	1.01E+07	4880340	48.53	2130709
SU06 Nx1	1.33E+07	7925689	59.6	3854062
SU06 Nx2	1.77E+07	10596063	59.97	4626186
SU06 Nx3	6.02E+06	3472761	57.66	1831557
SU09 Bx	9.47E+06	4209334	44.43	2409209
SU09 Nx1	1.86E+07	10337762	55.49	5685800
SU09 Nx2	1.46E+07	8535306	58.45	3762243
SU09 Nx3	1.87E+07	10148283	54.14	5726478
SU13 Bx	9.61E+06	4484764	46.65	2569407
SU13 Nx1	8.17E+06	4712170	57.69	2116199
SU13 Nx2	1.05E+07	5747588	54.88	3215960
SU13 Nx3	1.46E+07	8775348	59.96	3824543

SU16 Bx	8.05E+06	3329488	41.37	2155100
SU16 Nx1	9.62E+06	5472422	56.87	2673198
SU16 Nx2	1.42E+07	8285372	58.34	4104311
SU16 Nx3	8.23E+06	4639740	56.35	2122477
SU23 Bx	1.02E+07	4954929	48.66	2572098
SU23 Nx1	2.02E+07	11152738	55.21	6144702
SU23 Nx2	1.30E+07	7469297	57.28	4372123
SU23 Nx3	1.41E+07	8213089	58.45	4081282
SU25 Bx	8.99E+06	4069488	45.27	2396371
SU25 Nx1	8.25E+06	4660855	56.5	2769528
SU25 Nx2	1.61E+07	9086017	56.6	4092500
SU25 Nx3	1.80E+07	9056762	50.44	4586560
SU32 Bx	8.88E+06	3384069	38.1	2093500
SU32 Nx1	8.36E+06	4675505	55.96	2866189
SU32 Nx2	1.44E+07	7136930	49.42	4164458
SU32 Nx3	2.13E+07	12489618	58.63	5148200
SU36 Bx	1.01E+07	4632847	46.08	2540930
SU36 Nx1	1.96E+07	11464009	58.54	4763817
SU36 Nx2	1.12E+07	6844259	61.23	2635397
SU36 Nx3	1.34E+07	5843316	43.63	3375649
SU39 Bx	7.70E+06	3390124	44.04	2006866
SU39 Nx1	1.28E+07	7149482	56.02	4004067
SU39 Nx2	1.05E+07	6437080	61.11	2473941
SU39 Nx3	1.45E+07	8648897	59.76	3704362
SU46 Bx	1.10E+07	5187737	46.99	1828852

SU46 Nx1	1.19E+07	6854044	57.83	3085820
SU46 Nx2	1.61E+07	9633828	59.66	3306449
SU46 Nx3	1.31E+07	7703647	58.66	3893073
SU48 Bx	8.33E+06	4818707	57.82	2785646
SU48 Nx1	1.52E+07	9509365	62.46	3562889
SU48 Nx2	2.22E+07	13045505	58.89	5393978
SU48 Nx3	1.15E+07	6987142	60.56	3033454
SU54 Bx	1.12E+07	6919513	61.75	3012954
SU54 Nx1	1.09E+07	5633841	51.52	3389124
SU54 Nx2	1.19E+07	6824752	57.54	3382072
**Hypoxia set**				
**Sample name**	**Coverage**	**Mapped reads**	**Mapping percentage (%)**	**Library size**
RN318 Bx1	3.09E+07	15535782	50.31	4011496
RN318 Bx2	2.80E+07	14349433	51.22	4388699
RN318 Bx3	3.03E+07	17807770	58.68	8371972
RN318 Nx1	2.59E+07	17470943	67.58	7363073
RN318 Nx2	2.47E+07	14639628	59.18	4428042
RN318 Nx3	4.32E+07	25823984	59.79	3721210
RN319 Bx1	2.94E+07	15624532	53.14	8395422
RN319 Bx2	3.88E+07	20662427	53.19	9147450
RN319 Bx3	4.56E+07	28095154	61.65	9704698
RN319 Nx1	4.74E+07	32323988	68.2	8162019
RN319 Nx2	3.48E+07	22491140	64.7	10075238
RN319 Nx3	4.49E+07	21093255	47	6263883

Supplementary Table 7. Methylation differences following sunitinib therapy.

This table details the methylation differences in the 48 key ccRCC genes following patient treatment with sunitinib. 'Label' is the target gene label, 'logFC' is the fold change log, and, 'FDR' is the Benjamini-Hochberg adjusted *P*-value. *VHL* is the only target that has a false discovery rate (FDR) under the 0.1 significance level.

ID	Label	logFC	P.Value	FDR
7896829	*VHL*	-0.8734	0.00086	0.077
7757347	*PDGFB*	-0.6080	0.01733	0.373
7168671	*FLT1*	-0.5240	0.01744	0.373
7896831	*VHL*	-0.6491	0.01970	0.373
7497748	*CRTC1*	-0.5941	0.02312	0.373
7301263	*TSC2*	-0.5464	0.02902	0.373
7549722	*MTOR*	-0.4655	0.02882	0.373
7051648	*VEGFB*	0.48841	0.04208	0.454
8259570	*MET*	-0.4243	0.04538	0.454
7409596	*RPS6KB1*	-0.4416	0.06080	0.477
7892654	*PDCD1*	0.48114	0.06807	0.477
7514988	*EGLN2*	0.35425	0.06138	0.477
7497747	*CRTC1*	0.46051	0.08218	0.477
8199014	*PDGFA*	-0.4076	0.07849	0.477
7462032	*BCL2*	-0.4701	0.08224	0.477
7757350	*PDGFB*	0.44047	0.08659	0.477
7804829	*EPCAM*	-0.4760	0.09012	0.477

8259555	*MET*	0.38362	0.13240	0.585
8119354	*FLT4*	-0.3829	0.14980	0.585
8003316	*KIT*	-0.3852	0.15598	0.585
7964441	*PIK3CA*	0.35159	0.15038	0.585
7525064	*FLT3LG*	0.34752	0.16811	0.586
8100969	*PDGFRB*	0.37762	0.17304	0.586
8199005	*PDGFA*	-0.3154	0.12606	0.585
7168448	*FLT3*	0.34652	0.18558	0.585
8003445	*KDR*	0.34149	0.17925	0.585
7979502	*FGFR3*	-0.2877	0.14972	0.585
7896827	*VHL*	0.31411	0.20195	0.574
7051647	*VEGFB*	0.28838	0.20814	0.574
7892642	*PDCD1*	-0.2133	0.11484	0.574
8119356	*FLT4*	0.30784	0.24234	0.586
8322536	*TCEB1*	-0.3111	0.20831	0.586
6969832	*RET*	-0.3210	0.23228	0.586
7892656	*PDCD1*	0.26982	0.19302	0.586
6969782	*RET*	0.28235	0.28176	0.599
7549596	*MTOR*	0.27996	0.21950	0.599
7525066	*FLT3LG*	-0.2788	0.27822	0.606
8199013	*PDGFA*	0.25189	0.27282	0.606
7892652	*PDCD1*	0.27721	0.26008	0.606
8119355	*FLT4*	-0.2654	0.31343	0.606

7168447	*FLT3*	-0.2677	0.34623	0.606
7462028	*BCL2*	-0.2435	0.34789	0.606
7246263	*AKT1*	0.24431	0.35360	0.606
6991485	*PTEN*	-0.2394	0.34989	0.606
7168673	*FLT1*	-0.2430	0.37733	0.606
8307663	*FGFR1*	-0.2035	0.24162	0.606
8003312	*KIT*	0.22963	0.35977	0.629
7896828	*VHL*	-0.2064	0.28636	0.629
7757351	*PDGFB*	0.18731	0.32487	0.635
8307724	*FGFR1*	0.19462	0.42429	0.635
8307727	*FGFR1*	0.18324	0.37920	0.635
6969833	*RET*	0.18319	0.45964	0.635
7804792	*EPCAM*	-0.1744	0.47355	0.635
7168446	*FLT3*	0.18984	0.40386	0.635
6969783	*RET*	-0.1908	0.50366	0.635
7525063	*FLT3LG*	0.17452	0.51617	0.635
8199012	*PDGFA*	0.19330	0.46662	0.635
7514997	*EGLN2*	-0.1728	0.46263	0.635
8119353	*FLT4*	-0.1692	0.52323	0.635
7409659	*RPS6KB1*	0.16672	0.45664	0.635
7246265	*AKT1*	0.15913	0.53702	0.635
8367204	*CA9*	-0.1672	0.49071	0.635
8199004	*PDGFA*	-0.1615	0.33103	0.635

7462034	*BCL2*	0.11645	0.68596	0.635
8003444	*KDR*	0.09800	0.72513	0.635
7549723	*MTOR*	-0.0948	0.71864	0.635
7073658	*PDGFD*	0.09788	0.70967	0.635
6969780	*RET*	-0.1618	0.41049	0.635
7217306	*HIF1A*	-0.0470	0.86128	0.635
7615837	*CRTC2*	-0.1611	0.30947	0.635
7168672	*FLT1*	-0.0617	0.81151	0.635
7301323	*TSC2*	-0.1323	0.54176	0.635
8003314	*KIT*	0.03797	0.88807	0.635
7217305	*HIF1A*	-0.0749	0.76319	0.635
7892655	*PDCD1*	0.11393	0.57746	0.635
7892653	*PDCD1*	0.07355	0.76679	0.635
8437180	*KDM5C*	0.07358	0.78648	0.635
7301297	*TSC2*	0.00046	0.99829	0.635
7462033	*BCL2*	0.01272	0.95730	0.635
7246261	*AKT1*	0.04810	0.83986	0.635
7301260	*TSC2*	0.09567	0.61736	0.635
7301261	*TSC2*	0.10351	0.69703	0.635
7514998	*EGLN2*	-0.0400	0.87766	0.635
7514991	*EGLN2*	-0.1267	0.31202	0.635
7301262	*TSC2*	-0.1065	0.54358	0.719
7525065	*FLT3LG*	0.03312	0.85817	0.762

7979501	*FGFR3*	0.05789	0.76982	0.762
8259571	*MET*	0.02617	0.89036	0.762
7301319	*TSC2*	-0.0852	0.59300	0.762
7514990	*EGLN2*	-0.0193	0.90260	0.923

Supplementary Table 8. Sampling procedure methylation differences. This table shows the changes in methylation to the 48 key ccRCC genes induced following the hypoxic insult of renal artery ligation and warm ischaemia time prior to fresh sample acquisition (hypoxia set). 'Label' is the target gene label, 'logFC' is the fold change log, and, 'FDR' is the Benjamini-Hochberg adjusted *P*-value. There is no region that has a significant difference under the 0.1 FDR significance level.

**ID**	**Label**	**logFC**	**P.Value**	**FDR**
7892656	*PDCD1*	105.811	0.00091	0.102
7301262	*TSC2*	-0.8831	0.00434	0.243
7964441	*PIK3CA*	103.729	0.00772	0.288
7168674	*FLT1*	118.923	0.02861	0.534
7514991	*EGLN2*	-0.7320	0.02264	0.534
7514990	*EGLN2*	-0.7159	0.02739	0.534
7525063	*FLT3LG*	108.970	0.06153	0.721
7804829	*EPCAM*	-10.595	0.06421	0.721
8259571	*MET*	-0.6761	0.04633	0.721
8307726	*FGFR1*	0.88738	0.07060	0.721
8367204	*CA9*	-0.8619	0.07081	0.721
7462033	*BCL2*	-0.7359	0.07728	0.721
7549723	*MTOR*	-0.7478	0.10102	0.734
7757351	*PDGFB*	-0.6450	0.09063	0.734
7168672	*FLT1*	-0.8452	0.12999	0.734
7892655	*PDCD1*	0.56647	0.10756	0.734

8322536	*TCEB1*	-0.4791	0.09451	0.734
6969833	*RET*	-0.7575	0.16481	0.734
8367203	*CA9*	0.72068	0.17035	0.734
8259570	*MET*	-0.5480	0.11141	0.734
8037920	*VEGFC*	-0.6900	0.20309	0.764
7301261	*TSC2*	-0.5211	0.15990	0.764
8437198	*KDM5C*	-0.6446	0.26120	0.764
7757350	*PDGFB*	-0.5772	0.20595	0.764
7073658	*PDGFD*	0.61172	0.19525	0.764
8003445	*KDR*	-0.6358	0.25513	0.764
7462032	*BCL2*	-0.5432	0.19401	0.764
7896825	*VHL*	0.56909	0.24116	0.764
7051648	*VEGFB*	0.52596	0.19440	0.764
6969832	*RET*	-0.5401	0.24180	0.764
7409597	*RPS6KB1*	-0.5338	0.25917	0.764
7979499	*FGFR3*	0.58610	0.28178	0.764
8100970	*PDGFRB*	0.59250	0.29315	0.764
8199013	*PDGFA*	-0.4936	0.30608	0.764
7896824	*VHL*	0.48443	0.31532	0.764
7409596	*RPS6KB1*	-0.4146	0.17574	0.764
7514997	*EGLN2*	-0.4679	0.30962	0.764
7804792	*EPCAM*	-0.4896	0.36952	0.764
6969781	*RET*	-0.4993	0.35408	0.764

8151214	*VEGFA*	-0.4235	0.42781	0.764
8199014	*PDGFA*	-0.4614	0.38813	0.764
8259555	*MET*	0.42367	0.43322	0.764
8437199	*KDM5C*	0.41322	0.45585	0.764
7525066	*FLT3LG*	-0.3912	0.25718	0.764
8003316	*KIT*	-0.4322	0.45661	0.764
7462028	*BCL2*	-0.4071	0.41325	0.764
8003315	*KIT*	-0.4105	0.47274	0.764
7892653	*PDCD1*	-0.3945	0.34652	0.764
7462034	*BCL2*	0.39814	0.38585	0.764
8151218	*VEGFA*	-0.4252	0.39304	0.764
8307663	*FGFR1*	-0.3733	0.21251	0.764
7051647	*VEGFB*	-0.3971	0.38197	0.764
7168671	*FLT1*	-0.3624	0.48866	0.764
8307721	*FGFR1*	0.34187	0.51696	0.764
7804791	*EPCAM*	-0.3132	0.54336	0.764
7514998	*EGLN2*	0.36025	0.34044	0.764
8151216	*VEGFA*	-0.3094	0.58497	0.764
7246264	*AKT1*	-0.3214	0.54585	0.764
6991485	*PTEN*	-0.2883	0.56627	0.764
7246263	*AKT1*	0.26296	0.60768	0.764
7168446	*FLT3*	-0.2664	0.64261	0.764
7979502	*FGFR3*	-0.3513	0.40918	0.764

7896829	*VHL*	-0.3151	0.45941	0.764
8199012	*PDGFA*	-0.2327	0.64672	0.764
8151215	*VEGFA*	-0.1838	0.73300	0.764
6969780	*RET*	0.34418	0.26584	0.764
7497748	*CRTC1*	-0.3417	0.40745	0.764
8003314	*KIT*	-0.2539	0.61319	0.764
8003313	*KIT*	-0.1959	0.72609	0.764
8199005	*PDGFA*	-0.3398	0.31599	0.764
8119353	*FLT4*	-0.1446	0.80053	0.764
7896828	*VHL*	-0.3196	0.30065	0.764
7757347	*PDGFB*	-0.1800	0.73391	0.764
8003152	*PDGFRA*	-0.0789	0.88727	0.764
8003444	*KDR*	0.22166	0.67082	0.764
8100969	*PDGFRB*	-0.0211	0.97143	0.764
7979500	*FGFR3*	0.19962	0.69563	0.764
7896831	*VHL*	-0.1396	0.78078	0.764
8151219	*VEGFA*	-0.0630	0.90836	0.764
7246265	*AKT1*	-0.2265	0.62045	0.764
7301263	*TSC2*	-0.3009	0.46297	0.764
7896826	*VHL*	-0.1436	0.78964	0.764
7892654	*PDCD1*	-0.1956	0.67563	0.764
7217306	*HIF1A*	-0.2073	0.66184	0.764
7301297	*TSC2*	-0.3091	0.38483	0.764

7896830	*VHL*	-0.0410	0.93735	0.764
7409659	*RPS6KB1*	0.28260	0.34893	0.764
7217305	*HIF1A*	-0.3008	0.37012	0.764
7896827	*VHL*	-0.2087	0.64182	0.764
8003312	*KIT*	-0.0091	0.98571	0.764
8367202	*CA9*	0.04759	0.92458	0.764
7549596	*MTOR*	0.25400	0.50720	0.764
7979501	*FGFR3*	-0.2593	0.38937	0.764
7615837	*CRTC2*	-0.2733	0.25903	0.764
8307724	*FGFR1*	-0.1443	0.74090	0.764
8100971	*PDGFRB*	0.00495	0.99102	0.764
8119356	*FLT4*	0.19897	0.61613	0.764
8437180	*KDM5C*	0.06151	0.89120	0.764
7246261	*AKT1*	-0.2332	0.46178	0.764
7525064	*FLT3LG*	0.06932	0.86831	0.764
7301319	*TSC2*	-0.2439	0.37746	0.764
8307727	*FGFR1*	-0.1916	0.57223	0.764
7168448	*FLT3*	-0.0505	0.90151	0.764
7549722	*MTOR*	0.23538	0.44742	0.764
7525065	*FLT3LG*	-0.2182	0.49812	0.764
7497747	*CRTC1*	-0.1023	0.76212	0.764
7301323	*TSC2*	-0.0648	0.84666	0.764
7301260	*TSC2*	-0.1754	0.57051	0.764

8199004	*PDGFA*	0.17944	0.50971	0.764
7514988	*EGLN2*	0.17487	0.50461	0.764
7892652	*PDCD1*	0.01150	0.97008	0.764
7892642	*PDCD1*	0.17745	0.46380	0.764

Supplementary table 9. Driver mutation comparison between biopsy and nephrectomy samples. See figure 3a for corresponding bar charts. Comparison of SNV/indel mutation counts for all biopsy samples and all nephrectomy samples, representing the mean and SE across samples of that type (15 biopsy samples and 44 from the nephrectomies). Two-sided Wilcoxon rank sum test.

**Driver gene**	***P*-value**
ARID1A	0.594076
BAP1	0.5401108
CRTC2	0.4278332
FGFR1	0.4970986
FLT3	0.4278332
FLT4	0.594076
HIF1A	0.594076
HIF1AN	0.594076
KDM5C	0.8657747
MLH1	0.594076
MTOR	0.2430695
PBRM1	0.4603344
PDGFRA	0.0914805
PTEN	0.427922
SETD2	0.7310209
TSC2	0.4278332
VHL	0.5810538

Supplementary table 10. CNV counts for each sample on a per-patient basis. The mean CNV count is given for the nephrectomy samples. There was a mean change of 0.5 CNV counts between the biopsy and the mean of the nephrectomy samples for each patient. There was no significant difference in CNV count between the biopsy and mean of the nephrectomy samples (P=0.6; paired Student t-test). SU16 had no normal sample as such CNV analysis could not be performed. N/A-not applicable.

Patient	Biopsy	Nx1	Nx2	Nx3	Nx Average	Biopsy-Nx
SU05	5	3	1	0	1.3	3.7
SU06	0	2	0	1	1.0	-1.0
SU09	0	0	1	0	0.3	-0.3
SU13	0	0	0	0	0	N/A
SU23	1	0	1	3	1.3	-0.3
SU25	1	3	5	0	2.7	-1.7
SU32	0	1	2	1	1.3	-1.3
SU36	0	2	2	2	2.0	-2.0
SU39	1	0	0	1	0.3	0.7
SU46	1	0	0	0	0.0	1.0
SU48	0	0	2	1	1.0	-1.0
SU54	10	3	1	-	2.0	8.0

Supplementary table 11. Details at the individual patient level of the frequency of individual sample private mutations for biopsy samples (Bx) compared with the 3 nephrectomy samples (Nx). The mean number of private mutations for the nephrectomy samples are given. A rank of the sample type for which there is a higher number of private mutations is provided (biopsy compared with mean nephrectomy number of mutations).

Sample	**Bx private mutations**	Nx1 private mutations	Nx2 private mutations	Nx3 private mutations	**Mean Nx mutations**	**Bx vs average Nx mutation rank**
SU05	4	3	1	4	2.7	Bx
SU06	1	0	2	1	1.0	equal
SU09	0	1	4	0	1.7	Nx
SU13	4	1	4	0	1.7	Bx
SU16	1	0	0	1	0.3	Bx
SU23	1	1	1	1	1.0	equal
SU25	1	1	1	3	1.7	Nx
SU32	3	0	2	1	1.0	Bx
SU36	0	7	2	1	3.3	Nx
SU39	2	0	1	0	0.3	Bx
SU46	1	0	0	1	0.3	Bx
SU48	0	0	1	0	0.3	Nx
SU54	1	0	2		1	equal

Supplementary table 12. List of segments for which there is evidence of loss of heterozygosity (LOH). LOH occurring in all cells in a sample would result in germline heterozygous sites in the region covered by entirely reference or entirely alternate reads, which is evidenced by a B allele frequency (BAF: # alternate allele reads/total reads) of 0 or 1. An incomplete shift of the BAF away from 0.5 towards 0 or 1 indicates that not all cells in the sample have lost a copy of the locus. Y=LOH occurred in the indicated sample.

Patient	Chr	Start	End	Bx	Nx1	Nx2	Nx3	Length	Sites	Genes
SU32	chr1	11181327	27089446	Y				15908120	8	*MTOR*
SU48	chr1	11181327	153926078				Y	142744752	11	*MTOR*
SU09	chr3	37053568	178942431			Y		141888864	15	*MLH1, SETD2, BAP1, PBRM1, PIK3CA*
SU36	chr3	37053568	178942431	Y	Y	Y	Y	141888864	12	*MLH1, SETD2, BAP1, PBRM1, PIK3CA*
SU06	chr3	37053568	178947684	Y	Y	Y	Y	141894117	16	*MLH1, SETD2, BAP1, PBRM1, PIK3CA*
SU54	chr3	37053568	178947684	Y	Y			141894117	14	*MLH1, SETD2, BAP1, PBRM1, PIK3CA*
SU39	chr3	37067050	178942431		Y			141875382	15	*MLH1, SETD2, BAP1, PBRM1, PIK3CA*
SU46	chr3	37070106	47162661				Y	10092556	7	*MLH1, SETD2*
SU25	chr3	37070437	178942059		Y	Y	Y	141871623	10	*MLH1, SETD2, BAP1, PBRM1, PIK3CA*

SU48	chr3	47059323	52620347				Y	5561025	7	*SETD2, BAP1, PBRM1*
SU05	chr3	47059323	52643685		Y			5584363	8	*SETD2, BAP1, PBRM1*
SU23	chr3	47061194	178935943	Y	Y	Y	Y	131874750	13	*MLH1, SETD2, BAP1, PBRM1, PIK3CA*
SU25	chr5	149495253	180057356		Y		Y	30562104	14	*PDGFRB, FLT4*
SU06	chr7	538277	552046	Y	Y	Y	Y	13770	4	*PDGFA*
SU06	chr10	43572832	102295836		Y	Y	Y	58723005	7	*RET, PTEN, HIF1AN*
SU23	chr10	43610366	43613843	Y				3478	3	*RET*
SU23	chr13	28882948	28958955	Y				76008	3	*FLT1*
SU46	chr14	62203462	105242826				Y	43039365	6	*HIF1A, AKT*
SU46	chr16	2110571	2138422				Y	27852	10	*TSC2*
SU23	chr18	25543387	25585687	Y	Y			42301	3	*CDH2*

Supplementary table 13. Copy number variation (CNV) list for the 12 patients with an associated normal sample with which the ccRCC samples could be compared (SU16 did not have a normal sample). Estimated copy number is provided for each sample type per gene. A description of the CNV identified is provided as 'class'.

Patient	Gene	Chromosome	Start	End	Bx	Nx1	Nx2	Nx3	class
SU05	*TP53*	chr17	7572852	7579966	3				private_biopsy
SU05	*EPCAM*	chr2	47596466	47613775	1				private_biopsy
SU05	*TCEB1*	chr8	74858700	74872122	1				private_biopsy
SU05	*MET*	chr7	116339100	116436183		3			private_neph1
SU05	*KDM5C*	chrX	53221907	53254139	3	3			shared_biopsy_neph1
SU05	*RPS6KB1*	chr17	57970452	58025069	3	4	3		shared_biopsy_neph1/2
SU06	*FLT3,FLT1*	chr13	28578144	29069043		3			private_neph1
SU06	*CDH2,BCL2*	chr18	25531904	60985963		1		1	shared_neph1/3
SU09	*VEGFB*	chr11	64002851	64005906			1		private_neph2
SU23	*KDM5C*	chrX	53221907	53254139	3				private_biopsy

SU23	*VEGFB*	chr11	64002851	64005906				1	private_neph3
SU23	*FGFR3*	chr4	1800926	1809061				3	private_neph3
SU23	*TCEB1*	chr8	74858700	74872122			3	3	shared_neph2/3
SU25	*TCEB1*	chr8	74858700	74872122	1				private_biopsy
SU25	*CRTC1*	chr19	18794281	18888281		3			private_neph1
SU25	*MTOR,ARID1A*	chr1	11217169	27107285			3		private_neph2
SU25	*TP53*	chr17	7572852	7579966			3		private_neph2
SU25	*PIK3CA*	chr3	178916550	178952157			3		private_neph2
SU25	*VEGFB*	chr11	64002851	64005906		4	3		shared_neph1/2
SU25	*PDGFB*	chr22	39621669	39640001		3	3		shared_neph1/2
SU32	*PDGFRB*	chr5	149495157	149516621		3			private_neph1
SU32	*TCEB1*	chr8	74858700	74872122			3		private_neph2
SU32	*PDGFB*	chr22	39621669	39640001			3	3	shared_neph2/3

SU36	*TCEB1*	chr8	74858700	74872122		3			private_neph1
SU36	*PDGFA*	chr7	538073	558720			3		private_neph2
SU36	*MET*	chr7	116339100	116436183			3		private_neph2
SU36	*CDH2,BCL2*	chr18	25531904	60985963				1	private_neph3
SU36	*FGFR3*	chr4	1795546	1809061		3		3	shared_neph1/3
SU39	*PDGFA*	chr7	538073	557318				3	private_neph3
SU39	*TCEB1*	chr8	74858700	74872122	3			3	shared_biopsy_neph3
SU46	*TCEB1*	chr8	74858700	74872122	3			3	shared_biopsy_neph3
SU48	*HIF1A*	chr14	62162432	62213870				1	private_neph3
SU48	*TCEB1*	chr8	74858700	74872122			3	3	shared_neph2/3
SU54	*FLT3,FLT1*	chr13	28578144	29069043	3				private_biopsy
SU54	*CDH2,BCL2*	chr18	25531904	60985963	1				private_biopsy
SU54	*EPCAM*	chr2	47596466	47613775	3				private_biopsy

SU54	*CTLA4,PDCD1*	chr2	204732599	242801018	3				private_biopsy
SU54	*PDGFB*	chr22	39621669	39640001	1				private_biopsy
SU54	*TCEB1*	chr8	74858700	74872122	1				private_biopsy
SU54	*TP53*	chr17	7572852	7579966	4	4			shared_biopsy_neph1
SU54	*MET*	chr7	116339100	116436183	3	3			shared_biopsy_neph1
SU54	*PDGFA*	chr7	538073	552161	1	1	1		shared_biopsy_neph1/2
SU54	*CRTC1*	chr19	18853538	18888281	1		1		shared_biopsy_neph2
